# Evaluation of Complication Rates after Breast Surgery Using Acellular Dermal Matrix: Median Follow-Up of Three Years

**DOI:** 10.1155/2017/1283735

**Published:** 2017-06-12

**Authors:** Felix J. Paprottka, Nicco Krezdorn, Heiko Sorg, Sören Könneker, Stiliano Bontikous, Ian Robertson, Christopher L. Schlett, Nils-Kristian Dohse, Detlev Hebebrand

**Affiliations:** ^1^Department of Plastic, Aesthetic, Reconstructive and Hand Surgery, Agaplesion Diakonieklinikum Rotenburg, Elise-Averdieck-Straße 17, 27356 Rotenburg (Wümme), Germany; ^2^Harvard Medical School, Brigham and Women's Hospital, Department of Surgery, Division of Plastic Surgery, 75 Francis Street, Boston, MA 02115, USA; ^3^Department of Plastic, Reconstructive, Aesthetic and Hand Surgery, Alfried Krupp Krankenhaus, Hellweg 100, 45276 Essen, Germany; ^4^Department of Plastic, Aesthetic, Hand and Reconstructive Surgery, Hannover Medical School, Carl-Neubergstraße 1, 30625 Hannover, Germany; ^5^Department of Pathology, Agaplesion Diakonieklinikum Rotenburg, Elise-Averdieck-Straße 17, 27356 Rotenburg, Germany; ^6^Department of Surgery, Royal Brompton Hospital, Sydney St, London, UK; ^7^Department of Diagnostic and Interventional Radiology, University Hospital Heidelberg, Im Neuenheimer Feld 110, 69120 Heidelberg, Germany

## Abstract

**Introduction:**

Acellular dermal matrices (ADMs) are now commonly used for breast reconstruction surgery. There are various products available: ADMs derived from human (HADM), porcine (PADM), or bovine (BADM) sources. Detailed long-term follow-up studies are necessary to detect differences in complication rates between these products.

**Material and Methods:**

From 2010 to 2015, forty-one patients underwent 52 ADM-breast reconstructions in our clinic, including oncologic breast reconstructions and breast augmentation revisions (*n* = 52). 15x HADMs (Epiflex®/DIZG), 21x PADMs (Strattice®/LifeCell), and 16x BADMs (Tutomesh®/RTI Surgical) were implanted. Retrospective data collection with median follow-up of 36 months (range: 12–54 months) was performed.

**Results:**

Overall complication rate was 17% after ADM implantation (HADM: 7%; PADM: 14%; BADM: 31%). In a composite endpoint of complications and Red Breast Syndrome, a lower event probability was observed between BADMs, PADMs, and HADMs (44%, 19%, and 7%, resp.; *p* = 0.01 for the trend). Furthermore, capsular contracture occurred in 6%, more frequently as compared to the current literature.

**Conclusions:**

When ADM-based reconstruction is indicated, the authors suggest primarily the use of HADMs and secondary the use of PADMs. It is shown that BADMs have the highest complication probability within our patient cohort; nevertheless, BADMs convey physical advantages in terms of flexibility and better aesthetic outcomes. The indication for the use of ADMs should be filled for each case individually.

## 1. Introduction

An acellular dermal matrix (ADM) is a decellularized soft tissue material derived from a biological source. In breast surgery, ADMs have been used as an alternative to autologous myocutaneous flap grafts to bridge defects following aesthetic or oncoplastic breast reconstruction [1]. Advantages of this technique include improved implant stabilization as well as a decrease rate of capsular contracture [1]. Furthermore, ADMs can be used for reconstruction of the inframammary fold (IMF) in order to achieve an improved aesthetic outcome [2].

A variety of human derived ADMs (HADMs) are commercially available: Epiflex by DIZG (Berlin, Germany), Alloderm® by LifeCell Corporation (Bridgewater, NJ, USA), and DermaMatrix® by Synthes CMF (West Chester, PA, USA). However, Alloderm is not currently licensed for use in Germany. Strattice by LifeCell Corporation (Bridgewater, NJ, USA) is derived from porcine dermis/corium (PADM) and Tutomesh by RTI Surgical (Alachua, FL, USA) is derived from bovine pericardium.

Reported complications of ADM reconstruction techniques include skin necrosis, seroma, haematoma, infection, and Red Breast Syndrome (RBS). RBS is erythematous change occurring superficially to the area of ADM implantation without any specific signs of local infection; its mechanism is not well understood [3]. Few documented reports compare the long-term outcomes and associated risks of different types of ADMs [1, 4, 5].

The aim of this study is to assess the incidence of short- and long-term complications of implantation of various kinds of ADMs in our centre.

## 2. Material and Methods

Our analysis followed 41 female patients with a total of 52 ADM-based breast reconstructions between 2010 and 2015, using matrices such as Epiflex by DIZG (Berlin, Germany), Strattice by LifeCell Corporation (Bridgewater, NJ, USA), and Tutomesh by RTI Surgical (Alachua, FL, USA). Indications for reconstruction included secondary oncologic breast reconstructions (delayed), primary augmentations (in large breasts), or secondary breast augmentations (revision). In further detail, 15 HADMs (Epiflex), 21 PADMs (Strattice), and 16 BADMs (Tutomesh) were applied. Detailed information about the patient demographics with respect to type of ADM used is shown in Table 1. Indications for usage of ADMs within our patient cohort are listed in Table 2. Sixteen patients had a history of radiotherapy prior to ADM implementation; see Table 3. In all cases, ADMs were used in combination with subpectoral implant augmentation (Style 410 implants by Allergan); hereby these matrices were used to expand the pocket. Surgical drains were placed between the ADM and the implant. A single dose of intravenous antibiotics was administered intraoperatively.

Retrospective data analysis was performed using our hospital information system (HIS), median follow-up of 36 months (range: 12–54 months). Only patients with at least one year of follow-up time were included in our study. Patients were followed up at intervals of one week, two weeks, one month, three months, six months, and one year and then annually thereafter or if complications occurred. Assessment of short-term complications such as infection, seroma, haematoma, or skin necrosis and long-term complications such as capsular contracture (>Baker-St. II), implant malposition, or implant loss was made. Red Breast Syndrome (RBS) was the only complication occurring shortly after ADM usage in which no further medical treatment was indicated, though differential diagnosis might be challenging. Ultrasound imaging was performed in all patients with suspicion of RBS. Histopathological samples of the ADM implementation area were taken in four cases (*n* = 4).

### 2.1. Statistical Analysis

Continuous variables were expressed as median (range) and categorical variables as percentages (frequencies) if not other specified. Primary endpoints were skin necrosis, seroma, haematoma, infection, recurrence of capsular contracture, implant malposition, and implant loss. The composite endpoint included all complications as well as the Red Breast Syndrome. In order to analyze whether a trend exists regarding the endpoints between HADM, PADM, and BADM, Mantel-Haenszel Chi-Square was applied. Further, Chi-Square analysis allowed direct comparison between two kinds of ADMs. Statistical analysis was performed using the software SAS (Version 9.4, SAS Institute Inc., Cary, NC). A two-sided *p* value of <0.05 was considered statistically significant.

## 3. Results

All documented complications after implementations of ADMs occurring during the follow-up interval are demonstrated in Table 3. A relative reduction in probability for complications was observed between BADMs, PADMs, and HADMs, although statistical significance was not achieved (31%, 14%, and 7%, resp.; *p* = 0.07 for the trend). Similarly, the direct comparison between two kinds of the different ADM did not achieve statistical significance (all *p* > 0.08). Using a composite endpoint of complications and Red Breast Syndrome, the significant stepwise reduction was observed between BADMs, PADMs, and HADMs (44%, 19%, and 7%, resp.; *p* = 0.01 for the trend). In the direct comparison of two kinds of ADMs, HADMs demonstrated a significant lower probability for the composite endpoint as compared to BADMs (7% versus 44%, resp.; *p* = 0.02), while all other comparisons did not achieve statistical significance (all *p* > 0.10).

Three out of 15 patients, who were treated with Epiflex, required revision breast surgery: three patients received a mastopexy with contralateral breast refinement after 4, 7, or 8 months postoperatively. One month after breast reconstruction with ADM, an infection occurred in a patient with a history of radiotherapy; this was managed conservatively with antibiotics.

Five out of 21 patients, who received a Strattice implant, required revision surgery.

One patient needed revision surgery due to capsular contracture after 11 months postoperatively, and another with a history of radiotherapy had to be surgically revised due to skin necrosis after four months. One patient required ultrasound-guided drainage of a seroma after two months. After 5, 13, and 17 months postoperatively, three patients received a mastopexy with a contralateral mammary adaptation.

Four out of 16 patients, who were treated with Tutomesh, received a surgical revision.

one patient, with a history of chemotherapy, had a skin necrosis plus recurrence of capsular contracture after 6 months postoperatively, resulting in a DIEP-flap coverage. Another had a shell rupture of an implant after two months postoperatively, leading to a breast implant loss. After 10 months postoperatively, one patient with a recurrence of capsular contracture and a history of radiotherapy received a capsulotomy and implant exchange. After two months postoperatively, one patient with infection and loss of IMF underwent a surgical debridement followed by IMF-reconstruction and reaugmentation. In this group, there were no mastopexies with contralateral mammary adaptation during the given follow-up interval. Overall complications, after usage of all three given ADMs, are demonstrated in Table 4. In our study, an increased risk of general complications with 17% after ADM application could be shown. In further detail, complication risk for HADM (Epiflex) was 7% (1/15 patients), for PADM (Strattice) 14% (3/21 patients), and for BADM (Tutomesh) 31% (5/16 patients).

**Figure 1:**
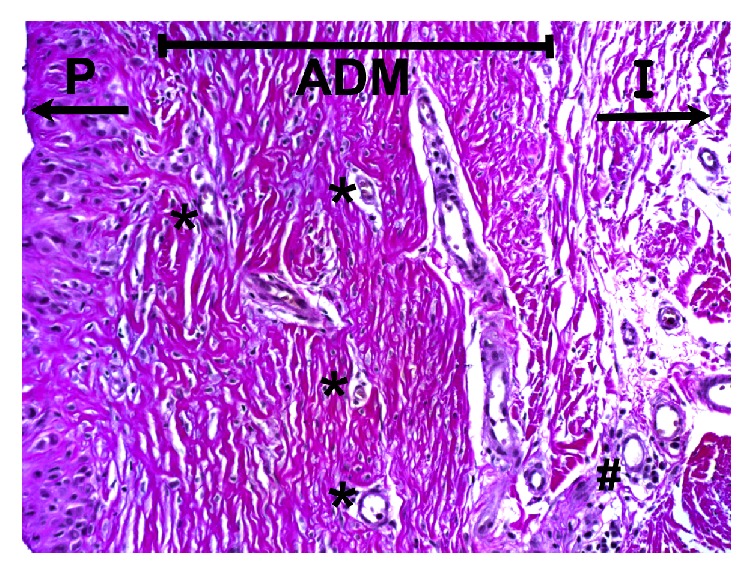
Histological slide within area of ADM implementation, H&E stain. Vascular invasion within the BADM (Strattice/LifeCell) after 6 months postoperatively. P = patient's side with breast tissue; ADM = acellular dermal matrix; I = implant side; *∗* = proliferation of new capillaries; # = foreign body response with soft tissue reaction. H&E stain; 200-fold microscopic magnification.

**Figure 2:**
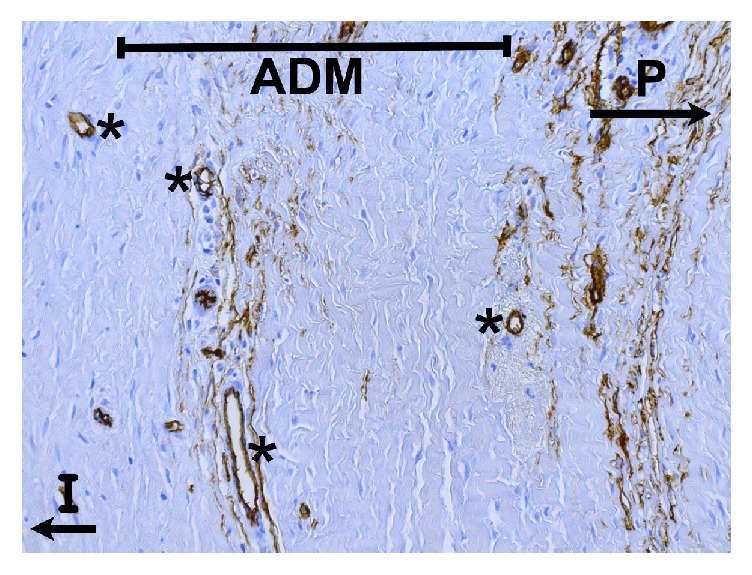
Histological slide within area of ADM implementation, CD34 stain. No histological evidence of capsular contracture in sample taken 6 months after BADM-implementation (Strattice/LifeCell). Immunohistochemistry showing strong expression of CD34 in endothelial cells within new grown vessels. P = patient's side with breast tissue; ADM = acellular dermal matrix; I = implant side; *∗* = new vessel formation. CD34 immunohistochemical staining; 200-fold microscopic magnification.

**Figure 3:**
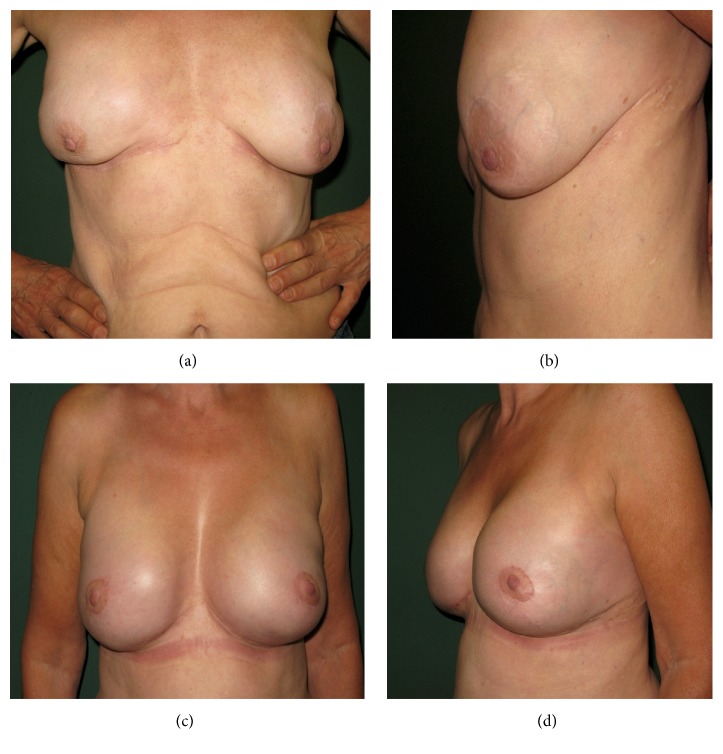
Case I: 57-year-old female patient suffering from implant malposition with lateral deviation, bottoming-out, rippling, ptosis with different nipple-areola complex (NAC) and IMF positions preoperatively; (a) frontal view; (b) lateral view; one-year postoperative results after BADM implantation (Strattice/LifeCell) with frontal (c) and lateral view (d).

**Figure 4:**
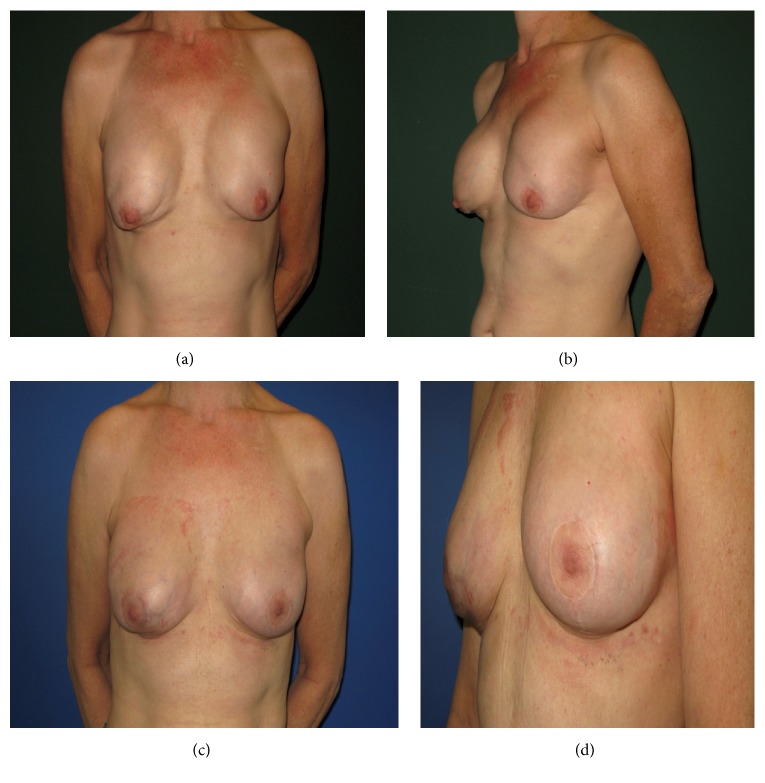
Case II: skin sparing mastectomy and breast augmentation performed in 54-year-old female patient with a history of breast carcinoma, complicated by shell rupture of the left-sided implant and subsequent breast-expander implantation; (a) frontal view; (b) lateral view; three-month postoperative results after HADM usage (Epiflex/DIZG) with frontal (c) and lateral view (d).

In four patients, who had undergone revision procedures, histopathologic biopsy samples could be taken within HADM-implementation zone (Figures 1 and 2). Furthermore, preoperative and postoperative images of two patient cases after ADM-breast reconstruction are demonstrated in Figures 3-4. 

## 4. Discussion

ADM-based techniques are now well established for breast reconstruction. There are various products available on the market: Epiflex is a cell-free dermis allograft, which is up to now the only licensed medicinal HADM product in German-speaking countries; Strattice is a sterile, acellular reconstructive tissue matrix, which is derived from porcine dermis; Tutomesh (BADM) is an avital, acellular, and xenogeneic membrane made from bovine pericardium. All manufacturers advertise their acellular matrices to rapidly integrate into the surrounding tissue without causing any immune response. Due to a special treatment of the human or animal source materials, cells, which may cause an autoimmune reaction, are washed out, leaving only a natural collagen membrane behind. But certainly, ADMs generated out of human, porcine, or bovine tissue might still have different qualities concerning tissue integration and postoperative outcomes.

According to the available literature, several studies have demonstrated reduction of capsular contracture [6, 7]. Long-term follow-up after primary breast augmentation without ADM implementation suggest the risk of capsular contracture formation is around 2%, 15%, and 19% after 3, 6, and 10 years, respectively [8–10]. Secondary augmentation revealed capsular contracture rates of 5%, 21%, and 29% after 3, 6, and 10 years, respectively [8–10]. The rate of capsular contracture occurrence after standard breast reconstruction with implants was 6%, 16%, and 25% after 3, 6, and 10 years, respectively [8–10]. Overall capsular contracture rate in our retrospective study was 6%, including one patient with a history of chemotherapy and one patient with a history of radiotherapy. Within the current literature, capsular contracture rates after ADM implantation differ from 0 up to 3.75% [6, 11–19].

The incidence of RBS in our cohort was 6% (3/52 patients), with one case in the Strattice and two cases in the Tutomesh group.

In comparison to previously published data, we demonstrate a higher overall complication rate after the implantation of ADM summing up to 17%. In detail, BADM (Tutomesh) had the highest complication probability with 31%, followed by PADM (Strattice) with 14%, and HADM (Epiflex) with 7%. A statistically increased complication probability was noted between the HADM and BADM group, if RBS was included to the other complications requiring further medical treatment. The inclusion of oncologic patients with breast reconstruction after/without radio- and/or chemotherapy may have resulted in greater heterogeneity and therefore increased complication rates.

Furthermore, it has already been shown that radio- or chemotherapy had an influence on implanted ADMs (Alloderm), resulting in a limited ADM modeling [20].

Salzberg et al. listed an overall complication rate of 3.9% in patients treated with prophylactic or oncologic SSM/NSM in combination with direct-to-implant immediate breast reconstruction using Alloderm (median follow-up: 28.9 months) [1]. In a meta-analysis, Newman et al. demonstrated a higher complication rate following HADM implementation with tissue expanders or permanent implants (12%) after therapeutic or prophylactic mastectomy and breast reconstruction [4]. Glasberg and Light published data in 2012, in which the complication rate after mastectomy and breast reconstruction with tissue expander was 6% using Strattice and 21% in patients with Alloderm [6]. Nevertheless, our presented data show an increased risk for complications after application of ADMs. In one of the largest studies, where Alloderm was used for breast reconstruction [1], rate of implant loss, skin necrosis, haematoma, capsular contraction, and infection were 1.3%, 1.1%, 0.4%, and 0.2%, respectively, compared with 2%. 4%, 6%, and 4%, respectively, in our study. However, there were no incidences of haematoma and implant malposition in our study, compared with 1.1% and 0.2%, respectively, in the studies by Salzberg et al. [1]. Seroma rate was 2% in our patient collective, whereas, in the study by Salzberg et al. [1], no information is given. Although Alloderm is made by the same manufacturer as Strattice, it is not available for purchase in Germany; therefore direct comparison of results should be interpreted with caution.

In a recent publication by Mendenhall et al., outcomes from time of tissue expander and HADM placement (Alloderm and DermaMatrix) to definitive reconstruction after simple and total skin and nipple-areola complex-sparing mastectomy were assessed [5]. Many of the treated breast cancer patients received chemo- or radiotherapy [5]. Overall complication rate in the Alloderm group was 33.6% and in the DermaMatrix group 38.8%, summing up to an all-breast complication rate of 36.2% [5]. 19.6% skin necrosis and 15.1% infection were the leading complications in this patient cohort [5]. Therefore, ADM implementation in patients receiving chemo-/radiotherapy seems to severely increase postoperative occurrence of complications [5]. Nevertheless, this data is not completely applicable to our patient cohort.

Other limitations of our study include the relative brevity of follow-up with a mean time period of 36 months. Many of the complications occurred early in the follow-up interval. Statements about the incidence of long-term complications such as capsular contractures are limited. Certainly, a larger patient collective and a longer follow-up interval are needed to obtain statistically significant results concerning complication rates.

As a result, indications for the use of ADMs should be considered on a case-by-case basis. Surely product costs may influence the surgeon's decision from time to time: from all three ADMs compared in our study, Strattice was the most expensive matrix and Tutomesh the one with the lowest price.

In our experience, ADMs are useful to correct these problems: loss of the IMF, implantation of high-volume implants, slight capsular contracture, bottoming-out, or other forms of implant malposition.

In this study, Epiflex (HADM) had the lowest complication rate, which may be due to allogenicity; nevertheless our detected total complication rate is greater than rates published within the available literature. In terms of preventing capsular contracture, thicker ADMs, such as Epiflex or Strattice, might be more appropriate for breast reconstruction. This effect could be explained due to prolonged vascular ingrowth time throughout the matrix and a delayed autoimmune response, caused by a longer isolation of the implant from the surrounding tissue. When aesthetic considerations are the greatest concern with loss of the IMF or bottoming-out of the implant, Tutomesh, which is made out of thinner bovine pericardium (offering a greater plasticity), may be favored. But an increased complication rate following BADM application has to be taken into account and should be discussed with the patient preoperatively.

## 5. Conclusions

Our retrospective study demonstrated that the use of HADMs is associated with the lowest complication risk (7%) among the three ADMs tested. In comparison with the available literature, the total complication rate of 17% was high. In our study, the capsular contracture rate after ADM reconstruction was 6% at a median follow-up interval of 36 months, that is also increased in comparison to other literature. The authors recommend a judicial use of ADM, taking into account costs, condition after radiotherapy, and uncertain long-term results. The flexible properties of BADMs have advantages in tissue handling and aesthetic properties but had the highest complication probability compared to the other subgroups within our study. Treatment of recurring capsular contracture calls for thicker ADMs such as Epiflex or Strattice. Overall, Epiflex (HADM) had the lowest complication rate within our study, whereas Tutomesh (BADM) showed the highest complication rate. In the case of loss of the IMF, large-volume implants, slight capsular contracture, bottoming-out, and implant malposition, the authors recommend in selected cases HADMs as primary and PADM as secondary treatment options. Due to the small amount of patients included in this retrospective study, a larger patient collective is urgently needed for further evaluation; therefore we suggest a prospective randomised study in order to receive more distinct results.

## Figures and Tables

**Table 1:** Patient collective with ADM-implementation.

ADM	Product	Number of ADM implants used	Number of treated patients	Avg. patient age (years)	Avg. follow-up time (months)
HADM	Epiflex/DIZG	15	12	46 (36–76)	40 (20–50)
PADM	Strattice/LifeCell	21	16	56 (44–66)	43 (30–54)
BADM	Tutomesh/RTI Surgical	16	13	53 (33–74)	20 (12–31)

Total	*All ADMs*	*52*	*41*	*52 (33*–*76)*	*36 (12*–*54)*

Listing of matrix, name of ADM-product and its fabricant, amount of breast reconstructions (BR)/augmentations with usage of ADMs, number of patients treated with ADMs, average patient age and range in years, and average follow-up time + range in months of the given patient collective, which received a breast reconstruction with ADM; HADM: human ADM, PADM: porcine ADM, and BADM: bovine ADM.

**Table 2:** Indications for ADM implementation.

ADM	Product	BR with ADM (*n*)	Oncologic indication		Aesthetic indication		Others^*∗*^
			BR with no capsular contracture^*∗*^	BR with capsular contracture^*∗∗*^	Primary augmentation^*∗*,*∗∗∗*^	Secondary augmentation after capsular contracture^*∗*^	
HADM	Epiflex/DIZG	15	1	3	9	2	0
PADM	Strattice/LifeCell	21	6	8	5	2	0
BADM	Tutomesh/RTI Surgical	16	2	5	3	2	4

Listing of kind of matrix used, name of product, number of breast reconstructions with certain ADMs, and indications for ADM usage (breast reconstruction (BR) with no capsular contracture, BR with capsular contracture, primary augmentation, secondary augmentation after capsular contracture). Oncologic patients made up 27% of the HADM group, 67% of the BADM group, and 44% of the BADM group; HADM: human ADM, PADM: porcine ADM, and BADM: bovine ADM; *∗* = no history of radiotherapy; *∗∗* = history of radiotherapy; *∗∗∗* = primary augmentation in cases with large breasts.

**Table 3:** Complications after ADM implementation.

ADM	Product	Complication rate^*∗*^	Skin necrosis	Seroma	Haematoma	Infection	Recurrence of capsular contracture	Implant malposition	Implant loss	RBS^*∗∗∗*^
HADM	Epiflex/DIZG	7%	—	—	—	1	—	—	—	—
PADM	Strattice/LifeCell	14%	1	1	—	—	1	—	—	1
BADM	Tutomesh/RTI Surgical	31%	1^*∗∗*^	—	—	1	2^*∗∗*^	—	1	2

Complications following ADM implantation for breast reconstruction with type of matrix, product, complications requiring further medical treatment (in %), infection, seroma, haematoma, skin necrosis, recurrence of capsular contracture, implant malposition, implant loss, and Red Breast Syndrome (RBS); HADM: human ADM, PADM: porcine ADM, and BADM: bovine ADM; *∗* = excluding occurrence of RBS; *∗∗* = one pat. with skin necrosis + recurrence of capsular contracture in two separate breasts; *∗∗∗* = not part of complications requiring further medical treatment.

**Table 4:** Overall complication probabilities for used ADMs.

ADM	HADM (Epiflex/DIZG), PADM (Strattice/LifeCell), BADM (Tutomesh/RTI Surgical)
Breasts (total)	52
Avg. follow-up time (in months)	36 (12–54)
Complications (total)	9 (17%)
Short-term complications	
Skin necrosis	2 (4%)
Seroma	1 (2%)
Haematoma	0 (0%)
Infection	2 (4%)
RBS^*∗*^	3 (6%)^*∗*^
Long-term complications	
Capsular contracture^*∗∗*^	3 (6%)
Implant malposition	0 (0%)
Implant loss	1 (2%)

Short-term (skin necrosis, seroma, haematoma, infection, and Red Breast Syndrome (RBS)) and long-term complications (capsular contracture, implant malposition, and implant loss) for all breasts with usage of ADMs (human ADM (HADM), porcine ADM (PADM), bovine ADM (BADM)), median follow-up time for all patients, total complications of all breasts being reconstructed with ADMs; *∗* = excluded from overall complications, which required further medical treatment; *∗∗* = >Baker-St. II.
