# Corrigendum to “Evaluation of Complication Rates after Breast Surgery Using Acellular Dermal Matrix: Median Follow-Up of Three Years”

**DOI:** 10.1155/2018/5731290

**Published:** 2018-07-11

**Authors:** Felix J. Paprottka, Nicco Krezdorn, Heiko Sorg, Sören Könneker, Stiliano Bontikous, Ian Robertson, Christopher L. Schlett, Nils-Kristian Dohse, Detlev Hebebrand

**Affiliations:** ^1^Department of Plastic, Aesthetic, Reconstructive and Hand Surgery, Agaplesion Diakonieklinikum Rotenburg, Elise-Averdieck-Straße 17, 27356 Rotenburg (Wümme), Germany; ^2^Harvard Medical School, Brigham and Women's Hospital, Department of Surgery, Division of Plastic Surgery, 75 Francis Street, Boston, MA 02115, USA; ^3^Department of Plastic, Reconstructive, Aesthetic and Hand Surgery, Alfried Krupp Krankenhaus, Hellweg 100, 45276 Essen, Germany; ^4^Department of Plastic, Aesthetic, Hand and Reconstructive Surgery, Hannover Medical School, Carl-Neubergstraße 1, 30625 Hannover, Germany; ^5^Department of Pathology, Agaplesion Diakonieklinikum Rotenburg, Elise-Averdieck-Straße 17, 27356 Rotenburg, Germany; ^6^Department of Surgery, Royal Brompton Hospital, Sydney St, London, UK; ^7^Department of Diagnostic and Interventional Radiology, University Hospital Heidelberg, Im Neuenheimer Feld 110, 69120 Heidelberg, Germany

In the article titled “Evaluation of Complication Rates after Breast Surgery Using Acellular Dermal Matrix: Median Follow-Up of Three Years” [1], there were errors, which should be corrected as follows.

(1) The title should be changed to “Evaluation of complication rates after breast surgery using two different kinds of Acellular Dermal Matrix and one Bovine Pericardium Collagen Membrane: median follow-up of three years.” 

(2) Tables 1, 2, and 4 along with their legends should be corrected as below.

(3) In the “Material and Methods,” the sentence reading “Sixteen patients had a history of radiotherapy prior to ADM implementation; see Table 3” should be changed to “Sixteen patients had a history of radiotherapy prior to ADM implementation; see Table 2.”

(4) In the “Discussion,” the sentence reading “Other limitations of our study include the relative brevity of follow-up with a mean time period of 36 months” should be changed to “Other limitations of our study include the relative brevity of follow-up with a median time period of 36 months.”

(5) The legends of Figures 3 and 4 should be corrected where “capsular contracture and” should be added to the legend of Figure 3 and “and capsular contracture, followed by” should be added to the legend of Figure 4. The corrected figures are as below.

## Figures and Tables

**Figure 3:**
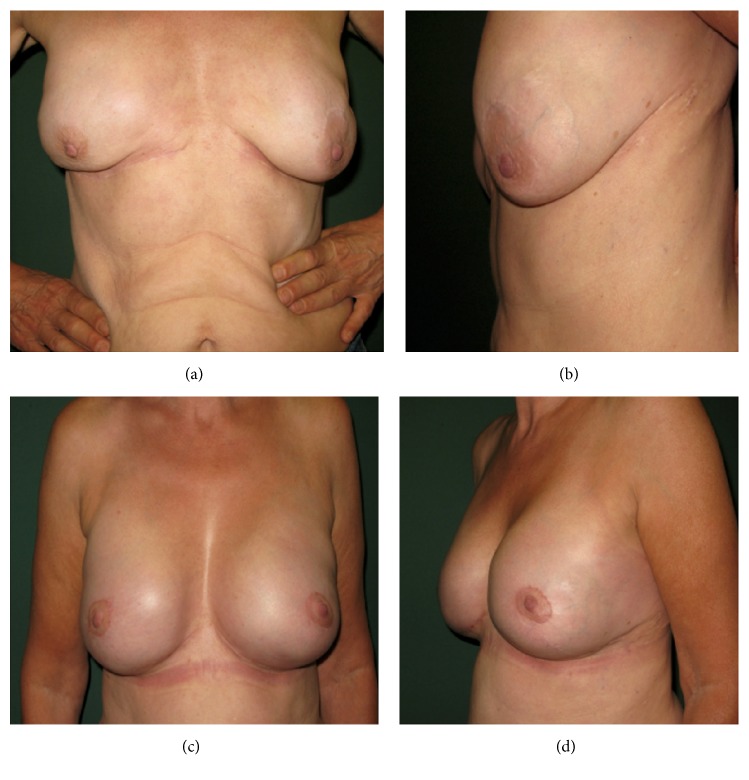
Case I: A 57-year-old female patient suffering from capsular contracture and implant malposition with lateral deviation, bottoming out, rippling, ptosis with different nipple-areola complex (NAC), and IMF positions preoperatively; (a) frontal view; (b) lateral view; one-year postoperative results after BADM implantation (Strattice/LifeCell) with frontal (c) and lateral view (d).

**Figure 4:**
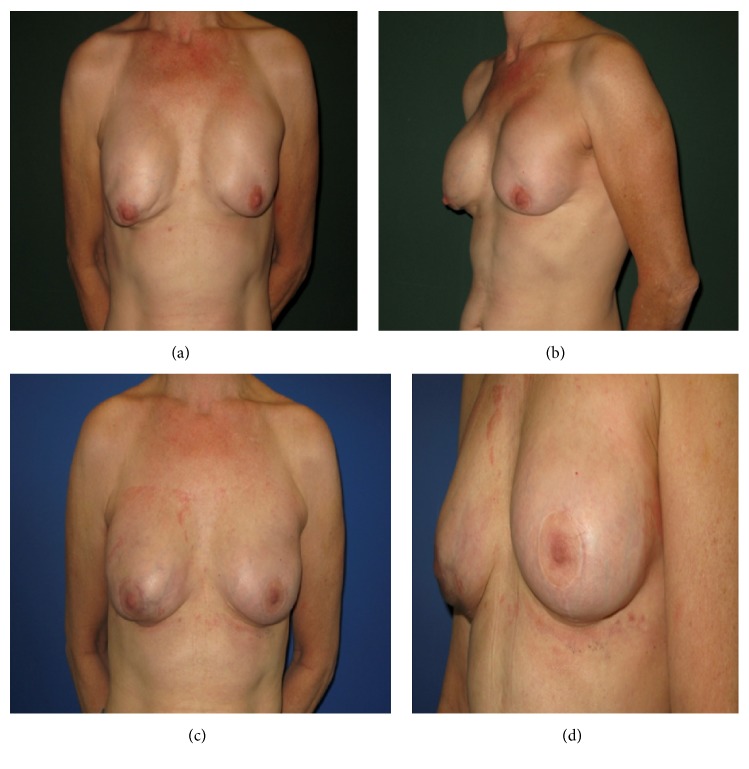
Case II: skin sparing mastectomy and breast augmentation performed in a 54-year-old female patient with a history of breast carcinoma, complicated by shell rupture of the left-sided implant and capsular contracture, followed by subsequent breast-expander implantation; (a) frontal view; (b) lateral view; three-month postoperative results after HADM usage (Epiflex/DIZG) with frontal (c) and lateral view (d).

**Table 1:** Patient collective with ADM-implementation.

**ADM**	**Product**	**Number of ADM implants used**	**Number of treated patients**	**Median patient age (years)**	**Median follow-up time (months)**
HADM	Epiflex® / DIZG	15	12	46 (36-76)	40 (20-50)
PADM	Strattice® / LifeCell	21	16	56 (44-66)	43 (30-54)
BADM	Tutomesh® / rti surgical	16	13	53 (33-74)	20 (12-31)

total	*all ADMs*	*52*	*41*	*52 (33-76)*	*36 (12-54)*

Listing of matrix, name of ADM-product and its fabricant, amount of breast reconstructions (BR)/augmentations with usage of ADMs, number of patients treated with ADMs, median patient age and range in years, and median follow-up time and range in months of the given patient collective, which received a breast reconstruction/augmentation with ADM; HADM: human ADM; PADM: porcine ADM; and BADM: bovine ADM.

**Table 2:** Indications for ADM implementation.

			**Oncologic indication**		***Aesthetic indication***		
**ADM**	**Product**	**BR with ADM (n)**	**BR with no capsular contracture ** ^**∗**^	**BR with capsular contracture ** ^*∗∗*^	**Primary augmentation ** ^**∗**^ **** ^*∗∗∗*^	**Secondary augmentation after capsular contracture ** ^*∗*^	**Secondary augmentation after aesthetic-related complications ** ^**∗**^ **** ^*∗∗∗∗*^
HADM	Epiflex® / DIZG	15	1	3	9	2	0

PADM	Strattice® / LifeCell	21	6	8	5	2	0

BADM	Tutomesh® / rti surgical	16	2	5	3	2	4

Listing of kind of matrix used, name of product, number of breast reconstructions with certain ADMs, and indications for ADM usage (breast reconstruction (BR) with no capsular contracture, BR with capsular contracture, primary augmentation, secondary augmentation after capsular contracture, or secondary augmentation after aesthetic-related complications including IMF-loss and insufficient implant coverage with breast tissue). Oncologic patients made up 27% of the HADM group, 67% of the PADM group, and 44% of the BADM group; HADM: human ADM, PADM: porcine ADM, and BADM: bovine ADM; *∗* denotes no history of radiotherapy; *∗∗* denote history of radiotherapy; *∗∗∗* denote primary augmentation in cases with large breasts; *∗∗∗∗* denote loss of IMF.

**Table 4:** Overall complication probabilities for used ADMs.

	**ADM**	HADM (Epiflex® / DIZG), PADM (Strattice® / LifeCell), BADM (Tutomesh® / rti surgical)
	**Breasts (total)**	52
	**Median follow-up time (in months)**	36 (12-54)
	**Complications (total)**	9 (17%)

**Short-term complications**	**Skin necrosis**	2 (4%)
	**Seroma**	1 (2%)
	**Haematoma**	0 (0%)
	**Infection**	2 (4%)
	**RBS** ^*∗*^	3 (6%)

**Long-term complications**	**Capsular contracture** ^*∗∗*^	3 (6%)
	**Implant malposition**	0 (0%)
	**Implant loss**	1 (2%)

Short-term (skin necrosis, seroma, haematoma, infection, and Red Breast Syndrome (RBS)) and long-term complications (capsular contracture, implant malposition, and implant loss) for all breasts with usage of ADMs (human ADM (HADM), porcine ADM (PADM), and bovine ADM (BADM)), median follow-up time for all patients, and total complications of all breasts being reconstructed with ADMs; *∗* denotes being excluded from overall complications and requiring further medical treatment; *∗∗* denote >Baker-St. II.
